# What counts as reliable evidence for public health policy: the case of circumcision for preventing HIV infection

**DOI:** 10.1186/1471-2288-11-34

**Published:** 2011-03-31

**Authors:** Reidar K Lie, Franklin G Miller

**Affiliations:** 1 Department of Bioethics, 10 Center Drive, Clinical Center NIH, Bethesda, MD 20892, USA

## Abstract

**Background:**

There is an ongoing controversy over the relative merits of randomized controlled trials (RCTs) and non-randomized observational studies in assessing efficacy and guiding policy. In this paper we examine male circumcision to prevent HIV infection as a case study that can illuminate the appropriate role of different types of evidence for public health interventions.

**Discussion:**

Based on an analysis of two Cochrane reviews, one published in 2003 before the results of three RCTs, and one in 2009, we argue that if we rely solely on evidence from RCTs and exclude evidence from well-designed non-randomized studies, we limit our ability to provide sound public health recommendations. Furthermore, the bias in favor of RCT evidence has delayed research on policy relevant issues.

**Summary:**

This case study of circumcision and HIV prevention demonstrates that if we rely solely on evidence from RCTs and exclude evidence from well-designed non-randomized studies, we limit our ability to provide sound public health recommendations.

## Background

Randomized controlled trials (RCTs) are widely regarded as the most scientifically rigorous method of evaluating the effect of interventions. However, there is an ongoing controversy over the relative merits of RCTs and non-randomized observational studies in assessing efficacy and guiding policy. According to the prevailing view, RCTs provide evidence far superior to observational studies. Evidence from observational studies by itself is reliable only if there is a very strong association between the intervention and the effect, such as a relative risk greater than 5 [1]. In the more usual case, one should rely on observational studies to evaluate efficacy only if for ethical or other reasons it is impossible to do an RCT. Evidence from observational studies may be useful to confirm that the results from RCTs apply in real life settings, but it can never, in the absence of a very strong association, provide conclusive evidence that an intervention causes an effect [2]. The main argument for this position is that adequate randomization is uniquely able to detect causation unbiased by confounding factors, because it balances known and unknown prognostic factors in the groups being compared.

Those who criticize this hierarchical view maintain that no one study design has an absolute advantage over any other design. On the whole, the results of any study, however well designed, cannot be taken by themselves to reveal the truth about the effect of an intervention. All studies are subject to confounding and bias. Accordingly, it is imperative to evaluate the totality of evidence, while trying to correct and adjust for confounding and trying to explain why there may be remaining differences in the results of existing studies [3].

Recently the debate has focused on whether investigators should include the results of observational studies in systematic reviews, when well designed RCTs are available. Those who argue for excluding observational studies from such reviews point out that the results from these studies are unreliable, by either under- or overestimating the true effect size [2,4]. Those who defend inclusion point out that when one focuses on well-designed observational studies, the effect sizes tend to be similar to RCTs, and that there is therefore no reason to exclude such evidence. Instead, in order to optimally inform practice and policy, it is important to include the totality of evidence from well-designed studies (randomized and observational) and to try to explain discrepancies between the included studies [3,5-7].

In this paper we will examine male circumcision to prevent HIV infection as a case study that can illuminate the appropriate role of different types of evidence for public health interventions. During the previous twenty years, multiple observational studies in the aggregate demonstrated a clear association between circumcision and HIV infection. Despite such consistent evidence, experts called for initiating RCTs because of the possibility of unknown confounders in the observational evidence. Only after the results of three RCTs were available was the public health community convinced that there was sufficient evidence to initiate provision of circumcision services in high prevalence areas [8-10].

We shall use this case to argue that ignoring or discounting observational evidence is a serious methodological error. To focus the analysis, we will compare two systematic reviews performed by the influential Cochrane Collaboration. The first assessed the evidence from observational studies; the second only assessed the evidence from recently completed RCTs. We limit ourselves to a discussion of these two reviews, recognizing that there are other reviews taking a different approach, such as [11]. By observational studies we mean studies where the intervention has not been assigned as a part of the research project, in particular not assigned by some random mechanism. The study subjects may be followed after they have selected the intervention, or the outcome may be measured long after the subjects selected their interventions.

Although we do not question the methodological advantages of RCTs, we argue that it is important to recognize that in the case of complex public health interventions the results from even well designed RCTs in isolation rarely provide enough evidence to guide sound public health policy. Only if we take into account the observational data do we have enough evidence to recommend circumcision as a public health policy to prevent HIV infection. Data from observational studies therefore need to be considered for inclusion and included when appropriate in systematic reviews of available evidence.

## Discussion

### The assumption that only RCTs can provide reliable evidence for policy

The Cochrane Collaboration is an international network dedicated to providing high quality systematic reviews of health care interventions. It is recognized as setting the standards for systematic reviews of evidence for the effectiveness of particular interventions. During the 1990s, based on data from multiple observational studies showing differences in HIV infection among circumcised and uncircumcised men, several experts recommended circumcision as a public health measure [12]. A Cochrane review published in 2003, however, concluded that there was no solid evidence that circumcision was effective:

Despite the positive results of a number of observational studies, there are not yet sufficient grounds to conclude that male circumcision, as a preventive strategy for HIV infection, does more good than harm. The results of current ongoing RCTs will need to be carefully considered before circumcision is implemented as a public health intervention for prevention of sexually transmitted HIV.... It would be prudent for consumers to await the findings of ongoing randomised trials before deciding on the balance between benefits and risks of male circumcision in the context of HIV infection [13] (p. 18).

The main reason for this negative conclusion was selection bias:

Selection bias was problematic in all studies, and results were potentially confounded by other risk factors for transmission of HIV, such as sexual behaviour and religion. Circumcised and uncircumcised groups (in cohort and cross-sectional studies) and HIV-positive and HIV-negative groups (in case-control studies) were seldom balanced for all or most of the ten risk factors that we identified as potential confounders prior to the quality assessment ... Statistical adjustments for measured confounding factors were made in 14 of the 35 included studies. The adjusted confounders differed across studies in number and type (p. 15).

At the time of this review, three RCTs were either in planning stages or had already started. The reviewers therefore saw no need to go into more detail with regard to an evaluation of the evidence [13]. In particular, they did not request more detailed data about the studies from the authors themselves in order to better judge whether selection bias was an issue in those studies that did not adjust for risk factors. But even if they had done so, it probably would not have altered their conclusion, given their stance that "observational studies are inherently limited by confounding which is unlikely to be fully adjusted for" (p. 2).

A second Cochrane review in 2009, in clear contrast to the negative conclusion in 2003, concludes quite forcefully that

Male circumcision can be considered as an effective measure to partly prevent HIV acquisition in heterosexual men. Current evidence is lacking for whether it also confers protection for women. Policy-makers can consider implementation of male circumcision as part of prevention measures if considered feasible and socially and culturally acceptable for local conditions [14] (p. 21).

It is noteworthy that the 2009 review was based *solely *on the evidence from the three RCTs that had been published in 2005 and 2007. Instead of adding the evidence from the RCTs to the existing review of the observational studies published in 2003, the 2009 review *replaced *the 2003 review as the authoritative review of the effectiveness of circumcision. It is possible that the authors assumed as background knowledge the observational studies examined previously, and therefore did not refer to them in the 2009 report. Another more likely explanation, however, is the view that once we have well-conducted RCTs we no longer need to consider evidence from observational studies because of its inherent unreliability. The dramatic shift from concluding that even prudent individuals should not consider circumcision, to an endorsement of circumcision as a public health intervention to prevent female to male transmission seems to presuppose the view that the RCT evidence is the *only *evidence that matters. We argue that this prevailing assumption cannot withstand critical scrutiny.

### Is circumcision causally related to risk reduction of HIV infection?

Both versions of the Cochrane reviews examine the causal role of circumcision in preventing HIV infection. The first concluded that observational studies have not demonstrated sufficiently that it is circumcision as such that prevents HIV infection, whereas the second concluded that RCTs have been able to identify this factor as contributing causally to HIV infection. The principal reason for these different conclusions is the impossibility of excluding confounders in observational studies, while RCTs are uniquely able to isolate the effect of the intervention of interest, in this case circumcision. Although this may be true for placebo-controlled drug trials where everything except the active substance is the same in the two comparison groups, this is generally not possible for an inherently complex intervention such as circumcision to prevent HIV infection, in which behavioral factors can interact with the medical procedure to influence the outcome under investigation. Obviously, the circumcision RCTs could not be conducted as a double-blind investigation. The lack of blinding with regard to the intervention received, as the Cochrane reviewers point out, is unlikely to affect the outcome assessment itself, as it is an objectively measured endpoint, HIV infection established by laboratory analysis. But since both the study personnel and the research participants would know who was circumcised or not, the RCT design cannot exclude differential distribution of behavioral interventions by the study personnel in the two groups (and thus the risk of HIV infection) independent of circumcision. This unequal distribution of behavioral interventions is independent of any effect circumcision may have on the risk behavior of the subjects themselves, as a result of circumcision.

Even though both the intervention (circumcision) and the outcome (HIV infection) can be standardized and objectively verified in an RCT, it is impossible to ensure that everything else of potential causal relevance in the two groups is exactly the same. For example, counseling is provided to everyone, but both those who are circumcised and the counselors know who are circumcised. Counseling may therefore be done differently in the two groups. Being members of the research team with an interest in proving the study hypothesis that circumcision prevents HIV, the counselors might more intensively counsel the circumcised group regarding risk behavior. Also, suspecting that the fact of circumcision might promote more risky behavior, the counselors might be inclined to deliver more forceful counseling to the circumcised group. Accordingly, it is possible that the specific counseling provided to the circumcised group causes the prevention of HIV infection, and not circumcision itself. We are not suggesting this as a plausible hypothesis for the RCT results; however, it does reflect a potential confound in the RCT evidence, which the Cochrane review did not address.

One also needs to monitor the effect of circumcision on the risk behavior of study participants. Changes in risk behavior following circumcision can complicate assessment of the causal impact of circumcision itself. A well designed RCT would, of course, monitor risk behavior in the two groups, which these trials did; however, they found variable effects on risk behavior in the two groups. The Ugandan trial did not report any difference in risk behavior in the two groups [10] whereas the South African trial reported increased risk behavior in the circumcised group [8]. Furthermore, the self-reported risk behavior might be unreliable, because a number of men became HIV positive in spite of reporting no risk behavior. In the Ugandan trial, for example 16 of the 67 infections occurred in men who reported no sex partners or 100% condom use [10]. It is therefore possible that there are unknown behavioral risk factors that are influenced by circumcision and differential reporting of risk behavior depending on group assignment that was not controlled for in the analysis. This is particularly a challenge because it is known from other studies that behavioral change is quite volatile, with a high level of risk behavior before circumcision, followed by excellent compliance after the procedure, but a return to previous levels of risk behavior some time after circumcision [15]. If differential distribution of risk behavior in the control and intervention groups counts as a decisive argument against observational studies, it should also count as a decisive argument against RCTs.

In the case of circumcision and HIV infection there is an additional, more complex type of causal relationship. Circumcision may be related to HIV infection via its effect on sexually transmitted infections (STIs). It is known that, in particular, ulcerative STIs such as syphilis may increase HIV infection. If circumcision reduces the risk of acquiring such STIs, this may explain the relationship between circumcision and HIV infection, even though circumcision has no direct, causal influence on HIV infection. It is therefore important to control for this risk factor when evaluating the causal role of circumcision in preventing HIV infection. Even if a well designed RCT demonstrates a clear difference in HIV infection between the two groups, this is not in and of itself a proof that circumcision has a causal effect independent of prevention of STIs. This factor needs to be controlled for in all types of studies. Only one of the RCTs did this by giving the numbers objectively diagnosed with STDs in the two groups [9].

The point here is not to express skepticism about whether circumcision has a causal role in HIV infection, nor to claim that confounding or bias are likely explanations of the protective effect demonstrated in the three RCTs, but to show that the prevailing approach to research evidence reflects an inherent bias against observational studies and in favor of RCTs. RCTs of complex interventions can rarely be designed with pristine experimental control; accordingly, they are subject to biases that need to be assessed. Nevertheless, factors that are considered decisive against the reliability of observational studies, often are not considered or are discounted when evaluating evidence from RCTs. In other words, the methodological limitations of observational studies are exaggerated at the same time that those of RCTs are underappreciated.

Importantly, the observational studies may help to correct the methodological limitations of RCTs for the evaluation of complex public health interventions. The rigor of observational studies of circumcision and HIV infection varies considerably. Although not all cross sectional studies have controlled for all possible behavioral confounders, many have controlled for quite a number of them, and in total, there is little evidence that there is an association between circumcision and behavioral risk factors that would explain the relationship between circumcision and risk of HIV infection. Further confidence comes from the several prospective studies where the number of HIV infections in two groups of initially HIV negative men, one circumcised and the other uncircumcised, were recorded [11]. These also confirm the hypothesis of a causal relationship between circumcision as such and HIV infection. These observational studies contain considerable data indicating that behavioral and other confounders cannot explain the relationship between HIV infection and circumcision. Accordingly, these data directly address the methodological questions regarding whether prevention of HIV infection by circumcision can be proved by RCTs. Rather than dismissing these data, a better strategy would be to conclude that we already know from the observational studies that there is a likely causal relationship between circumcision and HIV infection, and that the RCT evidence further confirms this. Whereas the RCT evidence by itself is not sufficient to establish a causal relationship, when combined with the observational data, there is proof beyond a reasonable doubt. In sum, failure to consider the relevant data from observational studies constitutes a serious methodological error.

### External validity: Will introduction of circumcision services reduce HIV infection?

When deciding policy, we not only need to know whether provision of circumcision protects against HIV infection (in the setting of an RCT). We also would want to know whether introduction of circumcision services will reduce HIV infection in the population. Even if we accept that circumcision has a causal role in HIV infection, we cannot necessarily conclude from this that implementing circumcision services will lead to a reduction in the incidence of HIV infection.

Based solely on evidence from the three RCTs it would be difficult to recommend a policy of promoting circumcision. We would have to assume that the situation of a population studied in the clinical trial, and the way circumcision was performed, is similar in all relevant respects to the situation when future populations decide to get circumcised. Specifically, we would have to assume that there are no behavioral risk factors for HIV infection that are associated with a decision to enter a circumcision trial versus a decision to utilize circumcision services promoted by public health authorities. This possible bias cannot be controlled for in any RCT. This is not merely a theoretical worry. A proportionally larger number of high risk individuals may decide to get circumcised if a nationwide program was introduced, who might subsequently increase risky behavior because they believe themselves to be protected. Additionally, sexually active HIV-positive men may decide to get circumcised if the perception is that circumcised men are less likely to be HIV-positive. Both of these factors could increase HIV infections and diminish or obliterate the positive causal role of circumcision in preventing HIV infection on a population level.

An additional weakness in the data from the RCTs is the lack of assurance that the circumcision method used in an RCT can be replicated in nationwide programs. Circumcision was provided in specialized clinics, with highly trained personnel, providing state of the art counseling. In particular, the trials included counseling to avoid any risk activity in the period right after circumcision due to the increase in risk of infection during this period. If circumcision is introduced on a large scale, it is highly likely that longer healing times after the procedure will be observed, and less adherence to reduction in risk behavior. It is also possible that circumcision might increase risk of HIV infection for women. Finally, if scaled up, the strict procedures for the performance of circumcision during a clinical trial setting may not be replicable. Less adherence to sterilization procedures may in fact increase the risk of HIV infection. Finally, the RCTs at the time they were reviewed by the Cochrane collaboration provided follow-up for no more than two years, and they were all stopped earlier than planned, providing even less data on longer term effects. All of this implies that we do not know, based on the evidence from only the RCTs, that there will be any long term, large scale positive effects of policies implementing circumcision at a population level.

If, on the other hand, we allow an equal consideration of the observational evidence, we may be more confident that introduction of circumcision services will reduce HIV infection. The cross-sectional studies provide long term data, and they focus on circumcision practices in settings that are closer to what one would reasonably expect in a nationwide program. While not completely reducing uncertainty, taking this evidence into consideration would strengthen the case for a policy promoting circumcision.

The results from the RCTs may overemphasize the effects we can expect in reducing HIV infection on a population level. Adult circumcision may not be as effective in preventing HIV in the general population because a relatively high number of adults will be infected before they are circumcised. The results from an adult HIV-negative population that is circumcised as adults in an RCT may therefore not be generalizable to what will happen if a public health circumcision program is introduced in an adult population because most HIV infections will occur before they are circumcised. If age of circumcision is an issue, therefore, the results from the three RCTs might overestimate the preventive effect of circumcision.

What all of this demonstrates is that if we *only *had the results from the three RCTs, we would have insufficient evidence to guide public policy. In this case, however, we do have substantial data from observational research that complement and strengthen the results from the RCTs, giving us sufficient confidence to recommend circumcision as a public health intervention to prevent HIV infection.

### Ignoring observational studies leads to suboptimal policy

The 2003 Cochrane review essentially concluded that no reliable evidence existed to infer that circumcision was causally responsible for preventing HIV infection and that it would therefore not be appropriate for individuals or policy makers to even consider introducing circumcision services. Based on the evidence available at that time, we think it would have been more appropriate to follow the recommendations published by experts a few years earlier. In 1999, Halperin and Baily argued that "The hour has passed for the international community to recognize the compelling evidence that show a significant association between lack of male circumcision and HIV infection" [12] (p. 1814). Based on the available evidence from observational studies, the authors recommended that one should both make this information available for individuals to make their own choices, but also introduce public health interventions aimed at increasing circumcision uptake. Public health authorities should

• Provide communities with accurate balanced information so that individuals can make informed choices

• Provide training and resources needed to offer safe, voluntary male circumcision in which pain is kept to a minimum

• Begin investigations of the feasibility of acceptable male-circumcision interventions in communities with high HIV and STD seroprevalence where circumcision has traditionally not been practiced.

While it may have been necessary to conduct RCTs in order provide more definitive evidence that could convince policy makers, more careful thought should have been given to how these trials should be designed and conducted, given the predictable uncertainties with regard to the additional evidence that one may obtain from RCTs pointed out above. Paradoxically, we are not able to provide much stronger recommendations than the above even *after *the results of the RCTs. In particular, we do not know if it would be worthwhile to establish widespread circumcision services even in high prevalence countries. Some key remaining uncertainties include lack of evidence regarding acceptability in different cultural groups, cost effectiveness, and applicability of the results to real life settings. Rather than conducting three trials with essentially similar cohorts and similar intervention strategies, a better strategy might have been to start one such trial and conduct a second "pragmatic" trial with an intervention strategy closer to what one could expect when circumcision services are implemented, by using for example cluster randomization by communities rather than individual randomization. In this second trial infection in women could also be monitored, as there is the possibility that male circumcision might lead to an increase in HIV infection in women. A cost-effectiveness analysis also could have been incorporated into such a study. There remain continued doubts about the cost-effectiveness of a circumcision program compared with other, established prevention programs, and there are doubts about whether circumcision will be as effective when highly active antiretroviral therapy is widely available for HIV [16]. Existing cost-effectiveness studies are modeling exercises and, although valuable, do not provide empirical data about how effective setting up circumcision services actually are in preventing HIV infection [17].

### Intersection of ethics and methodology

One should also consider the decision to stop all three trials early as a potential source of bias [18,19]. The prevailing ethics of clinical trials, oriented around the principle of equipoise, reinforces the biases against observational data and in favor of data from randomized trials. According to this principle there must be a state of equipoise regarding the benefit-to-risk ratio of the interventions under investigation in order to justify randomization, and trials must be stopped when this equipoise has been disturbed based on interim data [20]. Equipoise, suggesting indifference or at least uncertainty regarding the preventive efficacy of circumcision with respect to HIV, was possible at the outset of the three RCTs only on the basis of discounting the substantial observational data as not reflecting genuine knowledge. Once emerging data from each of these trials indicated statistically significant benefit from circumcision in reducing the risk of becoming HIV-positive, equipoise was disturbed, making it seem ethically imperative to stop the trials. However, the prior observational research supported the reasonable expectation that circumcision would prove to be effective. If it was ethical to commence the trial in the face of the observational data, why should it have been ethically necessary to stop them prematurely?

Stopping the three RCTs early diminished the knowledge regarding the effects of circumcision with respect to preventing HIV over time. Subjects entered the trial with the expectation that they would have a 50% chance of being circumcised and would be offered circumcision free of charge after completion of the trial if they were randomized to no circumcision. If it was reasonable and fair to invite men to participate on these terms, then we contend that it was also reasonable and fair to continue the trial to its planned endpoint. All the subjects had other means available to them to reduce their risk of becoming HIV-positive, including use of condoms and avoiding multiple sexual partners. The perceived imperative to stop the trials and offer circumcision to all the subjects confuses the individualistic, patient-centered ethics of medical care--the therapeutic obligation to offer optimal treatment--with the ethics of public health research, aimed at informing health policy [21].

The prevailing ethical perspective mirrors the prevailing epistemology. It is as if there was no knowledge at all about the efficacy of circumcision prior to undertaking RCTs and that definitive knowledge emerged as soon as a statistically significant difference in efficacy was detected in the RCT. Just as we need to correct epistemic biases that overvalue RCTs and undervalue well-designed observational research, so we need to correct ethical biases that needlessly impede the development of policy-relevant knowledge.

## Summary

This case study of circumcision and HIV prevention demonstrates that if we rely solely on evidence from RCTs and exclude evidence from well-designed non-randomized studies, we limit our ability to provide sound public health recommendations. One cannot, of course, generalize from one case only. RCTs, however, are increasingly being advocated for more complex public health interventions [22], and even for use in evaluation programs of anti-poverty programs by international organizations such as the World Bank [23]. Our analysis provides a reason to be skeptical of this trend, and it strengthens recent criticism of the applicability of randomized trials for public health in general [24] and for interventions in developing countries in particular [25]. More importantly, our case study illustrates the need to include all relevant studies in systematic analyses of evidence, and not focusing exclusively on RCTs that satisfy highly restrictive criteria. This is relevant for other interventions, as the ongoing controversy over the value of mammography screening to prevent breast cancer illustrates. In this case also the Cochrane group left out of their analysis a number of studies, thereby limiting the value of the review. In sum, all study methods have advantages and disadvantages with respect to rigor and relevance to guiding practice and policy. Despite general methodological advantages in determining causality, RCTs have distinctive limitations in developing policy-relevant knowledge. The hierarchical status that is commonly accorded to RCTs is unwarranted, especially in the field of public health. Combining evidence from well-designed RCTs and observational studies optimally informs public health policy.

## Competing interests

The authors declare that they have no competing interests.

## Authors' contributions

Both authors have been involved in the development of the ideas expressed in the paper and in the writing of the paper. Both authors approve of the final version of the paper.

## Pre-publication history

The pre-publication history for this paper can be accessed here:

http://www.biomedcentral.com/1471-2288/11/34/prepub
